# Health and Occupational Injury Experienced by Latinx Child Farmworkers in North Carolina, USA

**DOI:** 10.3390/ijerph17010248

**Published:** 2019-12-30

**Authors:** Thomas A. Arcury, Taylor J. Arnold, Sara A. Quandt, Haiying Chen, Gregory D. Kearney, Joanne C. Sandberg, Jennifer W. Talton, Melinda F. Wiggins, Stephanie S. Daniel

**Affiliations:** 1 Department of Family and Community Medicine, Wake Forest School of Medicine, Winston-Salem, NC 27157, USA; tjarnold@wakehealth.edu (T.J.A.); jsandber@wakehealth.edu (J.C.S.); sdaniel@wakehealth.edu (S.S.D.); 2 Department of Epidemiology and Prevention, Division of Public Health Sciences, Wake Forest School of Medicine, Winston-Salem, NC 27157, USA; squandt@wakehealth.edu; 3 Department of Biostatistics and Data Science, Division of Public Health Sciences, Wake Forest School of Medicine, Winston-Salem, NC 27157, USA; hchen@wakehealth.edu (H.C.); jtalton@wakehealth.edu (J.W.T.); 4 Department of Public Health, Brody School of Medicine, East Carolina University, Greenville, NC 27834, USA; kearneyg@ecu.edu; 5 Student Action with Farmworkers, Durham, NC 27705, USA; melinda.wiggins@duke.edu

**Keywords:** child labor, migrant and seasonal farmworkers, occupational health, agricultural health, health disparities, health equity, Latino/Hispanic

## Abstract

Children as young as 10 years old are hired to work on farms in the United States (U.S.). These children are largely Latinx. Using interview data collected from 202 North Carolina Latinx child farmworkers in 2017, this analysis documents the heath characteristics and occupational injuries of Latinx child farmworkers and delineates characteristics associated with their health and occupational injuries. Latinx child farmworkers include girls (37.6%) and boys (62.4%), aged 10 to 17 years, with 17.8% being migrant farmworkers. Three-quarters reported receiving medical and dental care in the past year. Respiratory (15.8%) and vision (20.3%) problems were prevalent. Girls more than boys, and younger more than older children had greater health service utilization. Occupational injuries were common, with 26.2% reporting a traumatic injury, 44.1% a dermatological injury, 42.6% a musculoskeletal injury, and 45.5% heat-related illness in the past year. Age increased the odds of reporting work injuries and heat-related illness, and being a non-migrant reduced the odds of reporting work injuries. These results emphasize the need for greater documentation of child farmworker occupational health and safety. They underscore the need to change occupational safety policy to ensure that children working in agriculture have the same protections as those working in all other U.S. industries.

## 1. Introduction

Children hired to work on farms in the United States (U.S.) constitute a vulnerable population [1]. Current U.S. regulations allow children as young as 10 years of age to be hired to work on farms not operated by their relatives [1], as long as they are engaged in nonhazardous jobs outside of school hours with parental permission on small farms to which Fair Labor Standard Act minimum wage requirements do not apply. Children aged 12 or 13 years can hold any nonhazardous farm job outside school hours with parental permission or on the same farm on which a parent is working. Those aged 14 or 15 years can hold any nonhazardous farm job outside school hours. Those aged 16 years or older can hold any farm job, hazardous or not, with unlimited work hours. Children of any age can do any farm job, hazardous or not, on farms operated by their relatives [2,3,4].

The exact number of children aged 10 to 17 years hired to work on U.S. farms is not known. The best available estimates, based on the National Agricultural Workers Survey [5] and the Childhood Agricultural Injury Survey [6], place the number of hired child farmworkers between 30,000 and 79,325 annually between 2005 and 2016. Most of these children are Latinx, either immigrants themselves or the children of immigrants.

Although children work on farms, agriculture is one of the most hazardous U.S. industries [7]. Agricultural work exposes children to environmental, mechanical, chemical, height, sharp tool, and sexual abuse hazards. As a result, children working on farms experience high rates of injury, illness, and mortality [8,9,10,11].

A few studies have documented the work and safety conditions of hired child farmworkers. Several indicate that child farmworkers generally do not receive required pesticide safety training [12,13,14,15,16], and that adherence to field sanitation regulations where they work is incomplete [12,13]. Child farmworkers often engage in hazardous tasks [17], but they often do not wear appropriate work clothing or personal protective equipment [12].

Little research documents the health and occupational injuries of Latinx child farmworkers [18]. Analyses report that a substantial percentage of hired child farmworkers experience non-fatal injuries [19], commonly report back pain [20], and commonly experience traumatic, dermatological and musculoskeletal injury [12]. Whitworth and colleagues [21] found high levels of neurotoxicity symptoms among hired child farmworkers.

Using data collected from North Carolina Latinx child farmworkers in 2017, this analysis has three aims. First, it describes general health characteristics of Latinx child farmworkers employed in North Carolina. Second, it describes the occupational injuries experienced in the past year by Latinx child farmworkers employed in North Carolina. Finally, it delineates personal and occupational characteristics associated with health characteristics and occupational injuries experienced in the past year by Latinx child farmworkers.

## 2. Materials and Methods

This analysis used data from the Hired Child Farmworker Study, a large community-based participatory research study conducted in North Carolina by investigators at Student Action with Farmworkers, Wake Forest School of Medicine, and East Carolina University. The investigators were supported by a child farmworker advisory committee [22] and a professional advisory committee. Arcury and colleagues [23] presented a detailed discussion of the overall study design. The Wake Forest School of Medicine Institutional Review Board approved the study protocol. Data for this analysis were collected from May through November 2017.

### 2.1. Participant Recruitment

Study participants were: (1) aged 10 to 17 years at recruitment; (2) self-identified as Latinx; (3) employed to do farm work in the past three months; and (4) fluent in Spanish or English. Both female and male child farmworkers were eligible. The study had no exclusion criteria.

Study participants were identified with the help of community partners who provide services to the North Carolina farmworker community. Interviewers developed lists of potential participants with the help of these community partners. They contacted children’s parents to explain the study, ensure the children met the inclusion criteria, discuss the monetary incentives for participation in the study, and obtain signed parental permission for the child’s participation. Interviewers then spoke with the potential participants, again reviewing the study, inclusion criteria, and incentive, and obtained signed assent. A few of the potential participants were “unaccompanied minors” individuals under age 18 years who do not live with a parent or legal guardian [1,24]. The Institutional Review Board approved recruiting these individuals without parental permission but with signed assent. Participants resided in 20 of North Carolina’s 100 counties. Because interviewers worked through community partners, the number of potential participants or their parents who refused to participate is not known.

### 2.2. Data Collection

The interview questionnaire included items taken from existing questionnaires and scales [12,25], with additional items developed by the investigators. The questionnaire was first developed in English. The study advisory committees reviewed the questionnaire content and the wording of specific items [22]. It was translated to Spanish, and then back translated to ensure item accuracy. The study advisory committees reviewed the English and Spanish versions. The field interviewers conducted pre-test interviews with youth who had formerly worked in agriculture. Questionnaire item wording was adjusted based on feedback received during pre-testing. The final interview questionnaire was designed to be completed within 45 min. Interviews were conducted with tablets using REDCap (Research Electronic Data Capture), a secure web-based system, to record data [26].

Bilingual individuals with knowledge of local farmworker communities from across NC recruited participants and conducted the interviews. All interviewers had experience with farmworkers through employment with organizations that provide services to farmworkers. Each completed an intensive training program that included a didactic component that discussed recruitment procedures, procedures for obtaining parental permission and participant assent, the interview content, and using the tablet. Interviewers were certified to contact participants after they successfully completed an audio-recorded or observed practice interview.

### 2.3. Measures

Measures for this analysis include participant personal and work characteristics, general health characteristics, and occupational injuries experienced in the past 12 months. Personal characteristics include gender (female, male), and age (in the categories 10–13 years, 14–15 years, 16–17 years). “Migrant worker” is a dichotomous indicator of whether the participant changes residence from state to state for agricultural employment. “Unaccompanied minor” is a dichotomous measure indicating that the participant lived with neither mother nor father. Work characteristics include “works with an adult relative” a dichotomous measure indicating whether the participant works with a parent, aunt, or uncle. The number of weeks worked in the last three months is in the categories: 1–2 weeks, 3–4 weeks, 5–7 weeks and 8–12 weeks.

Participant general health characteristics include the health services measures of whether the participant has a regular medical doctor, had a medical exam in the past year, and had a dental exam in the past year. Important participant health conditions included reporting respiratory problems, physician diagnosed respiratory problems, vision problems, physician diagnosed vision problems, hearing loss, physician diagnosed hearing loss, and loss of a digit or limb. Finally, participants reported if they had ever had a farm work injury that currently limits activities.

Occupational injuries are defined as injuries that participants reported having occurred while “doing farm work” in the past 12 months. These included “any injury” head blow resulting in being knocked unconscious; head blow resulting in dizziness, blurry vision, or not thinking clearly; eye injury; cut; skin rash; skin burn; sunburn; wrist pain; shoulder pain; ankle pain; and knee pain. For each injury, additional measures included whether the participant received medical care for the injury, whether the participant missed at least a half day of work or school due to the injury, and whether the participant was placed on light duty at work.

Summary measures include any traumatic injury (including head blows, eye injuries, and cuts); any dermatological injury (including skin rash, skin burn, and sunburn); and any musculoskeletal injury (including shoulder, ankle, and knee pain). Any work injury is a summary measure indicating if they had at least one of the specific injuries that occurred in the past year. Summary measures also indicate whether the participant received medical care, missed school or work, and was placed on light work duty for at least one specific injury that occurred in the past year.

Heat stress symptoms included reporting sudden muscle cramps, nausea or vomiting, hot, dry skin, confusion, dizziness, and fainting while working in extremely hot weather in the past year. Participants reporting any symptom were considered to have experienced heat-related illness.

### 2.4. Analysis

Frequency counts and percentages were used to describe the personal and work characteristics, general health characteristics, and occupational injuries of Latinx child farmworkers. The bivariate associations between personal and work characteristics and health outcomes and occupational injuries were examined using chi-square tests and Fisher’s exact tests when appropriate. Separate logistic regression models were fit for health services, health conditions, and occupational injuries. Odds ratios (ORs) and the associated 95% confidence intervals (CIs) were reported. All analyses were performed using SAS 9.4 (SAS, Cary, NC, USA). A *p* value of less than 0.05 was considered statistically significant.

## 3. Results

### 3.1. Personal and Occupational Characteristics

Two-hundred and two children completed interviews. Most (172, 85.1%) of the participants preferred being interviewed in English, with 30 (14.9%) preferring to be interviewed in Spanish. Seventy-six (37.6%) participants were female, and 126 (62.4%) were male (Table 1). They included 43 (21.3%) children aged 10–13 years, 64 (31.7%) aged 14–15 years, and 95 (47.0) aged 16–17 years. Thirty-six (17.8%) were migrant workers, and 18 (8.9%) were unaccompanied. Eight of the unaccompanied child farmworkers were migrants. Over half (121, 59.9%) of the children worked with an adult relative. Over the three months preceding their interview, 19.8% had done farm work for 1–2 weeks, while 35.2% had done farm work for 3–4 weeks, 27.7% for 5–7 weeks, and 17.3% for 8–12 weeks.

### 3.2. Health Characteristics

Most (65.4%) participants indicated that they had a regular medical doctor, that they had had a medical exam in the past year (76.6%), and that they had had a dental exam in the past year (71.8%) (Table 2). About one in six (15.8%) of the participants indicated that they had a respiratory problem, with 9.9% indicating that this problem was physician diagnosed; about one in five (20.3%) indicated that they had a vision problem, with 18.3% indicating that his problem was physician diagnosed. Four (2.0%) participants indicated they had hearing loss. One child reported losing a digit or limb. Seven (3.5%) participants indicated that a farm work injury currently limited their activities.

### 3.3. Personal and Work Characteristics Associated with Child Farmworker Health

More female than male Latinx child farmworkers had a regular medical doctor (72.4% versus 61.1%), had had a medical exam in the past year (80.3% versus 74.6%), and had had a dental exam in the past year (81.6% versus 65.9%), with differences for having had a dental exam being statistically significant (Table 3). More child farmworkers aged 10–13 years than those aged 14–15 years and 16–17 years had a regular medical doctor (75.6% versus 72.6% and 55.8%, respectively), had had a medical exam in the past year (88.9% versus 82.3% and 67.4%, respectively), and had had a dental exam in the past year (80.0% versus 72.6% and 67.4%, respectively), with these associations being statistically significant for having had a regular medical doctor and having had a medical exam in the past year. Significantly fewer migrant child farmworkers (38.9%) than non-migrant child farmworkers (71.1%) reported having a regular medical doctor. The number of weeks worked in the past three months had a significant inverse association with having a regular medical doctor; 70.0% of those working 1–2 weeks versus 42.9% of those 8–12 weeks reported having a regular medical doctor; 82.5% of those working 1–2 weeks versus 65.7% of those 8–12 weeks reported having had a medical exam in the past year. Child farmworkers reporting having a respiratory or vision problem, whether or not diagnosed by a physician, was not associated with gender, age, being a migrant worker, or working with adult relatives. However, number of weeks worked in the past three months had an inverse association with having respiratory problems (statistically significant), physician diagnosed respiratory problems, vision problems (statistically significant), and physician diagnosed respiratory problems. For example, 27.5% of those working 1–2 weeks versus 5.7% of those working 8–12 weeks reported respiratory problems; 35.0% of those working 1–2 weeks versus 14.3% of those working 8–12 weeks reported having vision problems.

The logistic regression analysis (Table 4) refines the associations of personal and work characteristics with health services and health conditions. Being male versus female significantly reduces the odds of having had a dental exam in the past year. Increasing age significantly reduces the odds of having had a medical exam in the past year, but not of having a regular medical doctor or of having had a dental exam. A greater number of weeks worked in the last three months is not associated with health services but does significantly decrease the odds of reporting any respiratory problem, any vision problem, and physician diagnosed vision problem.

### 3.4. Occupational Injuries in the Past 12 Months

Although 20 (9.9%) of the child farmworkers indicated that they had any farm work injury or illness in the past 12 months, this number increases substantially when they were asked about specific injuries (Table 5). No child reported being knocked out due to a blow to the head, two reported a blow to the head that resulted in being dizzy, blurry vison or not thinking clearly, and four reported eye injuries. On the other hand, 47 (23.3%) reported being cut, 40 (19.8%) reported a skin rash, 7 (3.5%) a skin burn, and 68 (33.7%) sunburn. Thirty-three (16.3%) reported wrist pain, 53 (26.2%) shoulder pain, 16 (7.9%) ankle pain, and 28 (13.9%) knee pain. Taken together, 26.2% reported a traumatic injury, 42.6% a dermatological injury, and 42.6% a musculoskeletal injury. Two-thirds (66.8%) report any work injury.

Few of the specific injuries resulted in medical care, missed school or work, or light duties. When taken together, for injuries occurring over the past 12 months, nine (4.5%) of these child farmworkers received medical care, 9 (4.5%) missed school or work, and 15 (7.4%) were put on light duty.

Almost half (45.5%) of the child farmworkers experienced heat-related illness while working over the past 12 months. Heat stress symptoms included dizziness (25.7%), sudden muscle cramps (21.8%), hot, dry skin (17.3%), nausea or vomiting (7.4%), confusion (5.0%), and fainting (1.5%).

### 3.5. Personal and Work Characteristics Associated with Occupational Injuries

Indicators of occupational injuries were not associated with the gender of the child farmworkers (Table 6). Several of the occupational injury indicators were significantly associated with child age and migrant status. More child farmworkers aged 16–17 years experienced each of the occupational injury indicators than those aged 14–15, and more of those aged 14–15 years experienced each of the occupation injury indicators than those aged 10–13 years, with these associations being statistically significant for any musculoskeletal injury, any work injury, and heat-related illness. For example, 20.0% of those 10–13 years old versus 38.7% of those aged 14–15 years old and 55.8% of those aged 16–17 years older reported a musculoskeletal injury. Of those 10–13 years old, 53.3% had any work injury, versus 64.5% of those aged 14–15 years old and 74.4% of those aged 16–17 years old. Similarly, more migrant child farmworkers reported each of the occupational injury indicators than did the non-migrant child farmworkers, with these associations being statistically significant for any traumatic injury (44.4% versus 22.3%), any dermatological injury (61.1% versus 40.4%), any musculoskeletal injury (63.9% versus 38.0%), and any work injury (86.1% versus 62.7%), as well as receiving medical care for the injury (16.7% versus 1.8%), missing school or work (3.9% versus 2.4%), and having light duty work (22.2% versus 4.2%) due to a work injury.

More of those child farmworkers who worked with an adult relative versus those who did not had any traumatic injury, dermatological injury, musculoskeletal injury, work injury, or heat stress symptom, as well as more of them receiving medical care, missing school or work, or having light duty due to a work injury. However, the only associations of working with an adult relative which reached statistical significance, were any musculoskeletal injury, with 48.8% of those working with an adult relative reporting any musculoskeletal injury versus 33.3% of those not working with an adult relative, and any work injury, with 72.7% of those working with an adult relative reporting any work injury versus 58.0% of those not working with an adult relative.

The logistic regression analysis (Table 7) emphasizes the associations of personal and work characteristics with occupational injuries and their consequences. Logistic regression analysis could not be completed for factors associated with receiving medical care or missing school or work due to any work injury because of the small number of cases. As in the bivariate analysis, gender did not significantly affect the odds of experiencing any of the injury measures or heat-related illness. Age increased the odds for each injury measure and heat-related illness; each year of increased age significantly increased the odds of any traumatic injury by 1.27 (95% Confidence Interval (95% CI) 1.04, 1.54), any dermatological injury by 1.24 (95% CI 1.04, 1.46), any musculoskeletal injury by 1.55 (95% CI 1.28, 1.89), any work injury by 1.37 (95% CI 1.14, 1.65), and heat-related illness by 1.41 (95% CI 1.18, 1.69). Being a non-migrant decreased the odds of experiencing injuries, but not heat-related illness; being a non-migrant worker decreased the odds of any traumatic injury by 0.30 (95% CI 0.12, 0.73), any dermatological injury by 0.35 (95% CI 0.14, 0.83), any musculoskeletal injury by 0.24 (95% CI 0.09, 0.63) and any work injury by 0.16 (95% CI 0.05, 0.55). Being a non-migrant also decreased the odds of light duty for any work injury by 0.11 (95% CI 0.03, 0.41; *p* = 0.0012). Working with an adult relative did not affect the odds of experiencing injury or heat-related illness. Increasing weeks worked had some effect on decreasing the odds of experiencing injuries. Compared to working 1–2 weeks, working 3–4 weeks decreased the odds of any traumatic injury by 0.44 (95% CI 0.18, 1.08), working 8–12 weeks decreased the odds of any musculoskeletal injury by 0.19 (95% CI 0.06, 0.60) and any work injury by 0.15 (95% CI 0.04, 0.50).

## 4. Discussion

Most Latinx child farmworkers in North Carolina experience an occupational injury each year, with two-thirds reporting a work injury in the past year and almost half reporting heat-related illness in the past year. Few of these child farmworkers received medical care for these work injuries. These injuries seldom caused them to miss school or work or require them to be placed on light duty at work. The occurrence of occupational injuries increased with age, so that those aged 16–17 years experienced more injuries in the past year than those aged 10–13 years. Migrant child farmworkers experienced more occupational injuries than did non-migrant children, and more migrant child farmworkers than non-migrants received medical care, missed school or work, and were put on light duty due to their injury. Working with an adult relative had some association with increased experience of occupational injuries (bivariate analysis). More weeks worked in the past three months had some association with decreased occupational injuries (logistic regression analysis). Child farmworker gender was not associated with the experience of occupational injury.

A few studies present information on occupational injuries experienced by child farmworkers. A pilot study of child farmworkers in North Carolina conducted in 2013 [12] found greater percentages of participants reporting any traumatic injury (60.9% versus 26.2% in the current study), any musculoskeletal injury (54.0% versus 42.6%), and any dermatological injury (72.4% versus 44.1%) than reported for the current study. Both studies found that few of the injured child farmworkers received medical care or missed school or work due to their occupational injuries.

Vela Acosta et al. [27] found that 9% of ninth-grade students enrolled in south Texas high schools and engaged in farm work reported a farm work-related injury. Shipp et al. [19] reported acute occupational injuries experienced by adolescent farmworkers, including those aged 18 and 19 years, who were attending Texas high schools. They found that 61 of 371 (16%) of their participants experienced an “acute injury” over a 9-month recall period, with seven (1.9%) of these adolescent farmworker receiving care from a medical care provider, and 40 (10.8%) receiving medical care from someone else (self, employer, friend). Twenty-one (5.7%) missed at least four hours of work due to their injury. Contrary to our finding of greater injury among older child farmworkers, they found that those aged less than 15 years and those aged 16 years, but not those aged 15 years and 18 years and older, had greater odds of experiencing an acute injury than those aged 17 years. Shipp et al. [20] also found that 15.7% of adolescent farmworkers reported occupational back pain, with increased odds of back pain among females, those with a history of back injury, feeling tense, stressed or anxious, tobacco use, and lifting heavy objects.

Any synthesis of these results is difficult. Only a small number of studies have focused on child farmworker occupational injuries. They differ in their sample design and composition, in the information they collected, and their definitions of injuries. However, a consistent finding across these studies is that child farmworkers commonly experience occupational injuries with little consistency in factors associated with the experience of these injuries. More focused research on child farmworkers is needed to determine how best to reduce the prevalence of these injuries.

The Latinx child farmworkers in this study reported high levels of health service utilization. Girls versus boys, young versus older, non-migrant versus migrant, and those working fewer weeks versus working more weeks reported greater health service utilization. Almost one in five reported respiratory or vision problems, with those working fewer weeks versus those working more weeks reporting more such health problems. The variation in health service utilization by gender (girls more than boys), age (younger more than older), migrant status (migrants less than non-migrants), and weeks worked (those working fewer weeks have greater utilization than those working more weeks) requires further investigation.

We found no studies reporting comparative information about child farmworker health service utilization or health conditions. Comparable information for adult farmworkers is available. Adult farmworker health service utilization is limited [28,29,30], making the high level of health service utilization among the child farmworkers participating in this study surprising. Potential explanations of the high utilization rates include the community-based recruitment process used by this study. Community-partner organizations, many of whom were clinics that provide health and dental care to the farmworker community, helped identify potential participants. These potential participants would have received care at these clinics. Many clinics providing care for the farmworker community conduct extensive outreach that includes health screenings; the child farmworkers may have interpreted such outreach care as medical and dental examinations. Finally, most of these children were attending school [23]; medical and dental exams might have been required for school or provided through the schools. This result warrants further analysis of child farmworker health service utilization.

Wheeze and other respiratory symptoms were common among adult Latinx farmworkers [31,32]. Compared to the 20% of child farmworkers who reported vision problems, Quandt et al. [33] found that 21.3% of adult, male Latinx farmworkers self-reported fair or poor vision, but only four wore corrective lenses or contacts.

The results of this analysis should be interpreted within its limitations. The sample was not randomly selected; organizations that serve the farmworker population assisted in recruiting participants known to them. However, no list of child farmworkers exists, and basing recruitment in schools would exclude migrants and those who might have left school. Recruiting child farmworkers across multiple counties enhances the samples’ representativeness. A response rate cannot be determined based on the recruitment strategy. The study focuses on Latinx child farmworkers living in one state, limiting the generalizability of the results. Finally, participants self-reported health and injury information, which may limit its accuracy.

## 5. Conclusions

Even with its limitations, this analysis provides important information needed to address the health and safety policy for child farmworkers. Although the participants in this study report high rates of health service utilization, these children experience high rates of work injury, including traumatic, dermatological, and musculoskeletal injuries, and heat-related illness symptoms. Migrant child farmworkers are at greater risk for all work injuries and those that require medical care than are non-migrant child farmworkers; fewer migrant child farmworkers versus non-migrants used health services. These results point to the need for greater documentation of child farmworker occupational health and safety. They also point to the need to change occupational safety policy to ensure that children working in agriculture have the same protections as those working in all other U.S. industries.

## Figures and Tables

**Table 1:** Participant personal and work characteristics, Latinx child farmworkers in North Carolina, 2017 (N = 202).

Personal and Work Characteristics	*n*	%
Gender		
Female	76	37.6
Male	126	62.4
Age (in years)		
10–13	43	21.3
14–15	64	31.7
16–17	95	47.0
Migrant Worker	36	17.8
Unaccompanied—lives with neither mother nor father	18	8.9
Works with adult relatives (including parents)	121	59.9
Work in last 3 months		
1–2 weeks	40	19.8
3–4 weeks	71	35.2
5–7 weeks	56	27.7
8–12 weeks	35	17.3

**Table 2:** General health characteristics, Latinx child farmworkers in North Carolina, 2017 (N = 202).

Health Characteristics	*n*	%
Health services		
Has regular medical doctor	132	65.4
Had medical exam in the past year	155	76.6
Had a dental exam in the past year	145	71.8
Health conditions		
Respiratory problems	32	15.8
Physician diagnosed	20	9.9
Vision problems	41	20.3
Physician diagnosed	37	18.3
Hearing loss	4	2.0
Physician diagnosed	2	1.0
Loss of digit or limb	1	0.5
Injury from farm work that currently limits activities	7	3.5

**Table 3:** Association of personal and work characteristics with health characteristics for North Carolina Latinx child farmworkers, 2017.

Personal and Work Characteristics	Health Services			Health Conditions			
	Has Regular Medical Doctor	Had Medical Exam in the Past Year	Had a Dental Exam in the Past Year	Respiratory Problems	Physician Diagnosed	Vision Problems	Physician Diagnosed
	*n* (%)	*n* (%)	*n* (%)	*n* (%)	*n* (%)	*n* (%)	*n* (%)
Gender							
Female	55 (72.4)	61 (80.3)	62 (81.6) *	10 (13.2)	6 (7.9)	20 (26.3)	17 (22.4)
Male	77 (61.1)	94 (74.6)	83 (65.9)	22 (17.5)	14 (11.1)	21 (16.7)	20 (15.9)
Age							
10–13	34 (75.6) *	40 (88.9) *	36 (80.0)	6 (13.3)	6 (13.3)	9 (20.0)	9 (20.0)
14–15	45 (72.6)	51 (82.3)	45 (72.6)	12 (19.4)	7 (11.3)	9 (14.5)	7 (11.3)
16–17	53(55.8)	64 (67.4)	64 (67.4)	14 (14.7)	7 (7.4)	23 (24.2)	21 (22.1)
Migrant Worker							
Migrant	14 (38.9) *	24 (66.7)	27 (75.0)	4 (11.1)	4 (11.1)	8 (22.2)	7 (19.4)
Non-migrant	118 (71.1)	131 (78.9)	118 (71.1)	28 (16.9)	16 (9.6)	33 (19.9)	30 (18.1)
Works with Adult Relative							
Yes	81 (66.9)	94 (77.7)	93 (76.9)	21 (17.4)	14 (11.6)	26 (21.5)	23 (19.0)
No	51 (63.0)	61 (75.3)	52 (64.2)	11 (13.6)	6 (7.4)	15 (18.5)	14 (17.3)
Amount of Work in Last Three Months							
1–2 weeks	28 (70.0) *	33 (82.5)	27 (67.5)	11 (27.5) *	6 (15.0)	14 (35.0) *	14 (35.0) *
3–4 weeks	54 (76.1)	60 (84.5)	56 (78.9)	13 (18.3)	8 (11.3)	16 (22.5)	13 (18.3)
5–7 weeks	35 (62.5)	39 (69.6)	41 (73.2)	6 (10.7)	4 (7.1)	6 (10.7)	6 (10.7)
8–12 weeks	15 (42.9)	23 (65.7)	21 (60.0)	2 (5.7)	2 (5.7)	5 (14.3)	4 (11.4)

* *p* < 0.05.

**Table 4:** Logistic regression analysis of personal and work characteristics with health characteristics for North Carolina Latinx child farmworkers, 2017.

**Personal and Work Characteristics**	**Health Services**												
	**Regular Medical Doctor**				**Medical Exam in the Past Year**					**Dental Exam in the Past Year**			
	**Odds Ratio (95% Confidence Interval)**			***p*-Value**	**Odds Ratio (95% Confidence Interval)**			***p*-Value**		**Odds Ratio (95% Confidence Interval)**			***p*-Value**
Gender (reference: female)													
Male	0.62 (0.32, 1.21)			0.1593	0.71 (0.34, 1.48)			0.3595		0.47 (0.23, 0.95)			0.0360
Age	0.84 (0.69, 1.01)			0.0581	0.73 (0.58, 0.91)			0.0053		0.92 (0.76, 1.11)			0.3663
Migrant Worker (reference: migrant)													
Non-migrant	4.25 (1.74, 10.41)			0.0015	1.88 (0.73, 4.87)			0.1914		0.80 (0.30, 2.14)			0.6532
Works with Adult Relative (reference: no)													
Yes	1.37 (0.69, 2.73)			0.3649	1.01 (0.48, 2.13)			0.9874		1.55 (0.78, 3.08)			0.2085
Amount of Work in Last Three Months (reference: 1–2 weeks)													
3–4 weeks	1.67 (0.67, 4.15)			0.2724	1.22 (0.41, 3.58)			0.7188		1.84 (0.75, 4.52)			0.1864
5–7 weeks	0.85 (0.34, 2.10)			0.7256	0.54 (0.19, 1.51)			0.2422		1.41 (0.56, 3.56)			0.4613
8–12 weeks	0.74 (0.26, 2.16)			0.5864	0.70 (0.21, 2.30)			0.5518		0.86 (0.29, 2.54)			0.7852
**Personal and Work Characteristics**	**Health Conditions**												
	**Respiratory Problems**						**Vision Problems**						
	**Any**		**Physician Diagnosed**				**Any**				**Physician Diagnosed**		
	**Odds Ratio (95% Confidence Interval)**	***p*-Value**	**Odds Ratio (95% Confidence Interval)**			***p*-Value**	**Odds Ratio (95% Confidence Interval)**		***p*-Value**		**Odds Ratio (95% Confidence Interval)**	***p*-Value**	
Gender (reference: female)	1.64 (0.71, 3.82)	0.2470	1.68 (0.60, 4.70)			0.3258	0.57 (0.27, 1.18)		0.1281		0.69 (0.32, 1.47)	0.3297	
Male													
Age	1.08 (0.87, 1.34)	0.4905	0.91 (0.71, 1.17)			0.4714	1.12 (0.92, 1.38)		0.2670		1.11 (0.90, 1.37)	0.3415	
Migrant Worker (reference: migrant)	1.13 (0.33, 3.91)	0.8414	0.66 (0.17, 2.51)			0.5433	0.66 (0.23, 1.86)		0.4307		0.60 (0.20, 1.78)	0.3583	
Non-migrant													
Works with Adult Relative (reference: no)	1.36 (0.58, 3.22)	0.4781	1.39 (0.47, 4.08)			0.5536	0.97 (0.44, 2.15)		0.9448		0.91 (0.40, 2.06)	0.8165	
Yes													
Amount of Work in Last Three Months (reference: 1–2 weeks)													
3–4 weeks	0.58 (0.23, 1.47)	0.2507	0.67 (0.21, 2.14)			0.5000	0.52 (0.21, 1.25)		0.1431		0.39 (0.16, 0.97)	0.0433	
5–7 weeks	0.32 (0.10, 0.96)	0.0426	0.46 (0.12, 1.81)			0.2656	0.20 (0.07, 0.58)		0.0035		0.20 (0.07, 0.59)	0.0036	
8–12 weeks	0.14 (0.03, 0.79)	0.0257	0.28 (0.04, 1.78)			0.1761	0.26 (0.07, 0.94)		0.0394		0.18 (0.05, 0.73)	0.0160	

**Table 5:** Farm work injuries in the past 12 months, Latinx child farmworkers in North Carolina, 2017 (N = 202).

Work Injuries	*n*	%
Any injury or illness	20	9.9
Received medical care	5	2.5
Missed school or work (at least half day)	8	4.0
Light duty at work	6	3.0
Head blow resulting in being knocked out	0	-
Head blow resulting in being dizzy, blurry vision, or not thinking clearly	2	1.0
Received medical care	1	0.5
Missed school or work (at least half day)	1	0.5
Light duty at work	1	0.5
Eye injury	4	2.0
Received medical care	0	-
Missed school or work (at least half day)	0	-
Light duty at work	1	0.5
Cut	47	23.3
Received medical care	3	1.5
Missed school or work (at least half day)	4	2.0
Light duty at work	5	2.5
Skin rash	40	19.8
Received medical care	2	1.0
Missed school or work (at least half day)	3	1.5
Light duty at work	3	1.5
Skin burn	7	3.5
Received medical care	0	-
Missed school or work (at least half day)	0	-
Light duty at work	0	-
Sunburn	68	33.7
Received medical care	1	0.5
Missed school or work (at least half day)	3	1.5
Light duty at work	1	0.5
Wrist pain	33	16.3
Received medical care	0	-
Missed school or work (at least half day)	0	-
Light duty at work	5	2.5
Shoulder pain	53	26.2
Received medical care	1	0.5
Missed school or work (at least half day)	1	0.5
Light duty at work	3	1.5
Ankle pain	16	7.9
Received medical care	0	-
Missed school or work (at least half day)	1	0.5
Light duty at work	2	1.0
Knee pain	28	13.9
Received medical care	1	0.5
Missed school or work (at least half day)	1	0.5
Light duty at work	2	1.0
Summary of work injuries		
Any traumatic injury	53	26.2
Any dermatological injury	89	44.1
Any musculoskeletal injury	86	42.6
Any work injury	135	66.8
Work injury consequences		
Received medical care for any work injury	9	4.5
Missed school or work (at least half day) due to any work injury	9	4.5
Light duty at work due to any work injury	15	7.4
Heat-related illness	92	45.5
Hear stress symptoms		
Dizziness	52	25.7
Sudden muscle cramps	44	21.8
Hot, dry skin	35	17.3
Nausea or vomiting	15	7.4
Confusion	10	5.0
Fainting	3	1.5

**Table 6:** Association of personal and work characteristics with occupational injuries in the past year for North Carolina Latinx child farmworkers, 2017.

Personal and Work Characteristics	Occupational Injuries					Consequences		
	Any Traumatic Injury	Any Dermatological Injury	Any Musculoskeletal Injury	Any Work Injury	Heat-Related Illness	Medical Care for Any Work Injury	Missed School or Work for Any Work Injury	Light Duty for Any Work Injury
	*n* (%)	*n* (%)	*n* (%)	*n* (%)	*n* (%)	*n* (%)	*n* (%)	*n* (%)
Gender								
Female	18 (23.7)	35 (46.1)	28 (36.8)	51 (67.1)	36 (47.4)	2 (2.6)	2 (2.6)	5 (6.6)
Male	35 (27.8)	54 (42.9)	58 (46.0)	84 (66.7)	56 (44.4)	7 (5.6)	7 (5.6)	10 (7.9)
Age (years)								
10–13	8 (17.8	15 (33.3)	9 (20.0) *	24 (53.3) *	13 (28.9) *	3 (6.7)	2 (4.4)	2 (4.4)
14–15	14 (22.6)	26 (41.9)	24 (38.7)	40 (64.5)	24 (38.7)	2 (3.2)	2 (3.2)	4 (6.5)
16–17	31 (32.6)	48 (50.5)	53 (55.8)	71 (74.4)	55 (57.9)	4 (4.2)	5 (5.4)	9 (9.5)
Migrant Worker								
Migrant	16 (44.4) *	22 (61.1) *	23 (63.9) *	31 (86.1) *	19 (52.8)	6 (16.7) *	5 (13.9) *	8 (22.8) *
Non-migrant	37 (22.3)	67 (40.4)	63 (38.0)	104 (62.7)	73 (44.0)	3 (1.8)	4 (2.4)	7 (4.2)
Works with Adult Relative								
Yes	36 (29.8)	57 (47.1)	59 (48.8) *	88 (72.7) *	56 (46.3)	7 (5.8)	7 (5.8)	11 (9.1)
No	17 (21.0)	32 (39.5)	27 (33.3)	47 (58.0)	36 (44.4)	2 (2.5)	2 (2.5)	4 (4.9)
Amount of Work in Last Three Months								
1–2 weeks	14 (35.0)	20 (50.0)	21 (52.5)	31 (77.5)	20 (50.0)	1 (2.5)	0	3 (7.5)
3–4 weeks	16 (22.5)	30 (42.3)	30 (42.3)	45 (63.4)	35 (49.3)	2 (2.8)	4 (5.6)	5 (7.0)
5–7 weeks	13 (23.2)	24 (42.9)	21 (37.5)	38 (67.9)	21 (37.5)	3 (5.4)	2 (3.6)	4 (7.1)
8–12 weeks	10 (28.6)	15 (42.9)	14 (10.0)	21 (60.0)	16 (45.7)	3 (8.6)	3 (8.6)	3 (8.6)

* *p* < 0.05.

**Table 7:** Logistic regression analysis of personal and work characteristics with occupational injuries in the past year for North Carolina Latinx child farmworkers, 2017.

Personal and Work Characteristics	Any Traumatic Injury		Any Dermatological Injury		Any Musculoskeletal Injury		Any Work Injury		Heat-Related Illness	
	Odds Ratio (95% Confidence Interval)	*p*-Value	Odds Ratio (95% Confidence Interval)	*p*-Value	Odds Ratio (95% Confidence Interval)	*p*-Value	Odds Ratio (95% Confidence Interval)	*p*-Value	Odds Ratio (95% Confidence Interval)	*p*-Value
Gender (reference: female)	1.41 (0.69, 2.85)	0.3435	0.95 (0.52, 1.74)	0.8654	1.94 (0.99, 3.79)	0.0521	1.20 (0.62, 2.35)	0.5872	0.90 (0.49, 1.67)	0.7478
Male										
Age	1.27 (1.04, 1.54)	0.0191	1.24 (1.04, 1.46)	0.0137	1.55 (1.28, 1.89)	<.0001	1.37 (1.14, 1.65)	0.0008	1.41 (1.18, 1.69)	0.0001
Migrant Worker (reference: migrant)	0.30 (0.12, 0.73)	0.0082	0.35 (0.14, 0.83)	0.0176	0.24 (0.09, 0.63)	0.0035	0.16 (0.05, 0.55)	0.0034	0.62 (0.26, 1.48)	0.2840
Non-migrant										
Works with Adult Relative (reference: no)	1.42 (0.68, 2.99)	0.3518	1.21 (0.64, 2.27)	0.5610	2.01 (1.01, 3.99)	0.0463	1.76 (0.89, 3.47)	0.1031	1.11 (0.58, 2.10)	0.7542
Yes										
Amount of Work in Last Three Months (reference: 1–2 weeks)										
3–4 weeks	0.44 (0.18, 1.08)	0.0730	0.65 (0.29, 1.45)	0.2929	0.53 (0.22, 1.27)	0.1547	0.41 (0.16, 1.04)	0.0606	0.93 (0.41, 2.12)	0.8672
5–7 weeks	0.47 (0.18, 1.19)	0.1121	0.64 (0.27, 1.49)	0.2985	0.40 (0.16, 1.00)	0.0512	0.49 (0.18, 1.33)	0.1621	0.47 (0.20, 1.14)	0.0952
8–12 weeks	0.32 (0.10, 1.03)	0.0556	0.39 (0.14, 1.13)	0.0843	0.19 (0.06, 0.60)	0.0049	0.15 (0.04, 0.50)	0.0021	0.51 (0.18, 1.46)	0.2082
